# Mitigating Scar Tissue Formation in Tendon Injuries: Targeting HMGB1, AMPK Activation, and Myofibroblast Migration All at Once

**DOI:** 10.3390/ph16121739

**Published:** 2023-12-17

**Authors:** Jianying Zhang, Roshawn Brown, MaCalus V. Hogan, James H-C. Wang

**Affiliations:** 1 MechanoBiology Laboratory, Department of Orthopaedic Surgery, University of Pittsburgh, E-1640 BST, 200 Lothrop Street, Pittsburgh, PA 15213, USA; jianying@pitt.edu (J.Z.); brownr24@upmc.edu (R.B.); hoganmv@upmc.edu (M.V.H.); 2 Department of Bioengineering, University of Pittsburgh, Pittsburgh, PA 15213, USA; 3 Department of Physical Medicine and Rehabilitation, University of Pittsburgh, Pittsburgh, PA 15213, USA

**Keywords:** metformin, tendon injury, scar tissue

## Abstract

Tendon injuries, while prevalent, present significant challenges regarding their structural and functional restoration. Utilizing alpha-smooth muscle actin (α-SMA)-Ai9-scleraxis (Scx)-green fluorescent protein (GFP) transgenic mice, which exhibit both Scx (a tendon cell marker) and α-SMA (a myofibroblast marker), we explored the effects of metformin (Met) on tendon healing, repair, and its mechanisms of action. Our findings revealed that intraperitoneal (IP) injections of Met, administered before or after injury, as well as both, effectively prevented the release of HMGB1 into the tendon matrix and reduced circulating levels of HMGB1. Additionally, Met treatment increased and activated AMPK and suppressed TGF-β1 levels within the healing tendon. Tendon healing was also improved by blocking the migration of α-SMA^+^ myofibroblasts, reducing the prevalence of disorganized collagen fibers and collagen type III. It also enhanced the presence of collagen type I. These outcomes highlight Met’s anti-fibrotic properties in acutely injured tendons and suggest its potential for repurposing as a therapeutic agent to minimize scar tissue formation in tendon injuries, which could have profound implications in clinical practice.

## 1. Introduction

Tendons are well-organized, fibrous, musculoskeletal tissues that are highly susceptible to acute injuries. Injured tendons exhibit poor healing, often accompanied by scarring. This scarring represents an overgrowth of fibroblastic cells within the connective tissue network that impairs the function of affected tissues and organs [1]. In the United States, over 300,000 tendon repairs are performed annually [2]. These repairs often necessitate lengthy periods of rehabilitation due to the tendons’ limited intrinsic regenerative capacity. In addition, compromised tendons are prone to re-injury [3]. Existing treatments, primarily physical therapy and surgery, frequently fall short of restoring full tendon function. Consequently, tendon injuries and scar tissue formation significantly impair mobility and quality of life. Addressing scarless tendon healing has become a key challenge for both orthopedic surgeons and researchers alike.

Scar formation is characterized by the excessive accumulation of extracellular matrix (ECM) components, mainly produced by myofibroblasts, in response to injury. These changes are associated with the expression of α-smooth muscle actin (α-SMA) [4,5]. The growth factor TGF-β1 plays a crucial role in pathological fibrosis as shown by several preclinical studies across different organs [6,7]. 5′-AMP-activated protein kinase (AMPK) is a cellular energy sensor [8] and can protect against fibrosis in several organs including the liver, heart, lung, and kidney [9,10,11,12] by inhibiting the TGF-β1 signaling pathway [13,14].

Inflammation can significantly impact the late stage of tissue repair, potentially leading to scar formation. High mobility group box 1 (HMGB1), a ubiquitous protein present in all cells and a potent inflammatory mediator, has been implicated in the pathogenesis of various inflammatory diseases, including tendon overuse injuries such as tendinopathy. This has been demonstrated by multiple studies, including our own [15,16,17,18]. The role of HMGB1 in fibrotic diseases is also well-established. The release of HMGB1 following tissue injury is associated with fibrotic processes in systemic sclerosis and liver, renal, and pulmonary fibrosis [19,20,21]. Research has shown that HMGB1 can promote scar formation, while its blockade results in a drastic reduction in scarring [22,23,24]. These studies underscore the significance of HMGB1 as a modulator of scar formation and suggest that blocking HMGB1 may have therapeutic value in preventing and treating fibrotic diseases.

Metformin (Met), an FDA-approved drug commonly prescribed for type 2 diabetes, is known to inhibit HMGB1 [25], and has emerged as a potential candidate for preventing and treating fibrosis. The anti-fibrotic effects of Met have been demonstrated in various fibrotic pathologies, including pulmonary [26,27,28], cardiac [29], joint capsular [30], and liver fibrosis [31].

Met works by activating AMPK and reducing the levels of TGF-β1, a key fibrotic growth factor [32,33,34,35]. In previous research, we have shown that the intraperitoneal (IP) injection of Met can inhibit the development of tendon inflammation and degeneration by blocking HMGB1 release [18,36]. Thus, Met may also be efficacious in reducing scarring in injured soft tissues such as tendons, by blocking fibrosis and regulating inflammation at the same time.

However, it is not known whether Met is beneficial in reducing scar tissue formation in wounded tendons. In this study, we investigated the effect of Met on the healing of wounded tendons using α-SMA-Ai9-Scx-GFP transgenic mice that express both Scx (a tendon cell marker) and α-SMA (myofibroblast marker). We report that Met injection before and/or after tendon injury was able to improve tendon healing and mitigate scar tissue formation by blocking HMGB1 release, activating AMPK, and inhibiting myofibroblast migration to the wound area.

## 2. Results

### 2.1. Met Injection Blocks HMGB1 Release and Reduces HMGB1 Levels

**Figure 1:**
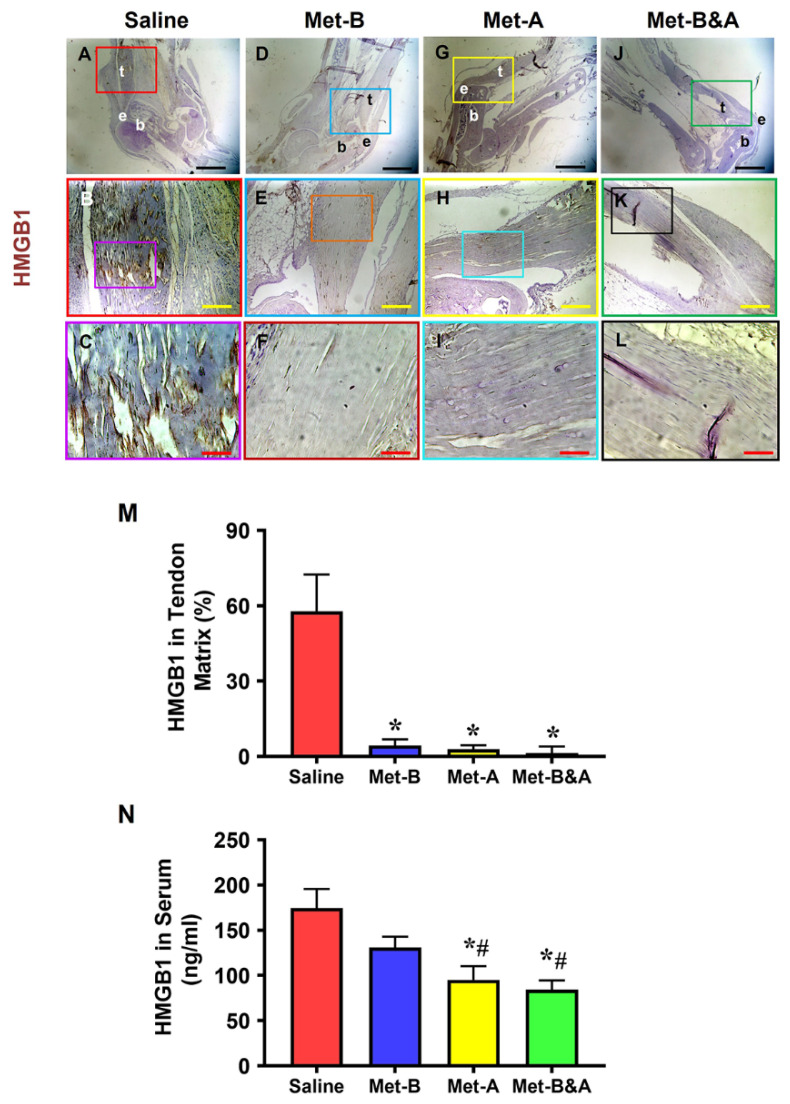
**Met injection inhibits the release of HMGB1 from cell nuclei to the tendon matrix and reduces HMGB1 levels in the sera of mice.** Immunohistochemical (IHC) staining results for HMGB1 revealed elevated HMGB1 levels in the tendons of the saline group (**A**–**C**). However, in Met-injected mice (**D**–**L**), there was a notable reduction, evidenced by markedly lower HMGB1 staining levels. Semi-quantification demonstrated that over 57% of cells in saline-treated tendons displayed positive HMGB1 staining (**M**), while fewer than 5% of cells in Met-injected tendons exhibited positive HMGB1 staining (**M**). * *p* < 0.01 compared to saline. As determined by ELISA, HMGB1 levels in the sera of mice at 4 weeks post-wounding decreased in a Met-treatment-time-dependent manner (**N**); * *p* < 0.01 compared to saline; # *p* < 0.05 compared to Met-B. Each of the small boxes, presented in four different colors (**A**,**D**,**G**,**J**) is correspondingly enlarged in the larger boxes (**B**,**E**,**H**,**K**). Similarly, each of the small boxes, presented in four different colors (**B**,**E**,**H**,**K**) is correspondingly enlarged in the larger boxes (**C**,**F**,**I**,**L**). Black bars: 1 mm; yellow bars: 200 µm; red bars: 50 µm. **Met-B**: Met-Before; **Met-A**: Met-After; **Met-B&A**: Met-Before-and-After; **t**: tendon; **b**: bone; **e**: enthesis.

Elevated levels of HMGB1 were evident in the tendons of mice receiving IP injections of saline (Figure 1A–C). In contrast, IP Met injection effectively suppressed HMGB1 release, leading to significantly reduced HMGB1 staining in Met-treated mice (Figure 1D–L). The semi-quantitative analysis revealed that 57% of cells in saline-treated tendons exhibited positive HMGB1 staining (Figure 1M), whereas less than 5% of cells in Met-injected tendons displayed such staining (Figure 1M).

The concentrations of HMGB1 in the sera of mice subjected to IP Met injection exhibited a time-dependent decrease. This difference was particularly pronounced in the **Met-A** and **Met-B&A** groups, compared to both the saline and **Met-B** groups (Figure 1N).

### 2.2. Met Injection Increases and Activates AMPK

**Figure 2:**
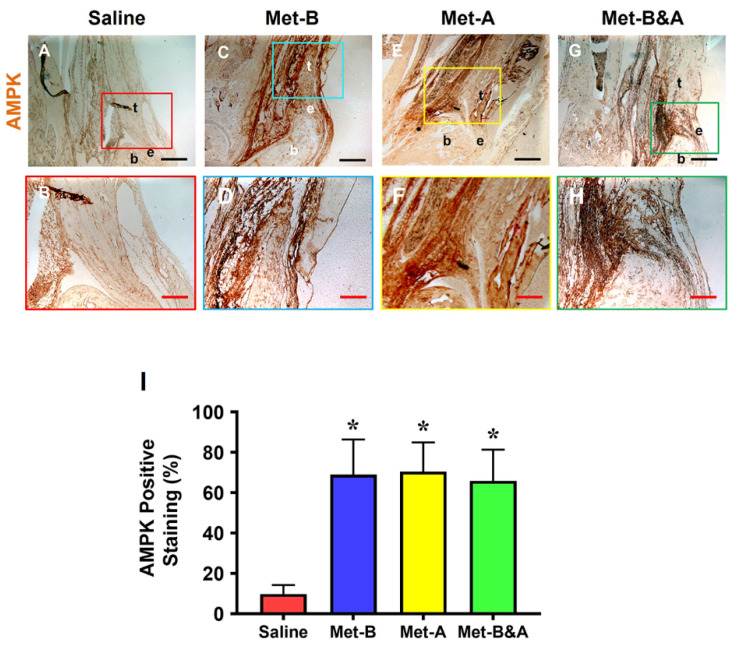
**Met injection increases AMPK.** The IHC staining showed that AMPK levels were increased in the wound tendons of Met-injected mice (brown areas, (**C**–**H**)) compared to the saline-injected group, which showed minimal AMPK staining (**A**,**B**). Semi-quantification results indicated that over 65% of tendon cells in the mice with Met injection were positively stained with AMPK. However, less than 10% of tendon cells in the mice with saline injection were positively stained with AMPK (**I**). * *p* < 0.01 compared to saline. Each of the small boxes, presented in four different colors (**A**,**C**,**E**,**G**) is correspondingly enlarged in the larger boxes (**B**,**D**,**F**,**H**). Black bars: 500 µm; red bars: 200 µm. **Met-B**: Met-Before; **Met-A**: Met-After; **Met-B&A**: Met-Before-and-After; **t**: tendon; **b**: bone; **e**: enthesis.

Met injection significantly increased AMPK levels in mouse tendon tissue (Figure 2C–H), while minimal staining was observed in the wounded tendons of mice receiving IP saline injections (Figure 2A,B). The semi-quantitative analysis revealed that over 65% of tendon cells in Met-injected mice exhibited positive staining for AMPK, whereas fewer than 10% of tendon cells in the saline-injected group displayed such staining (Figure 2I).

**Figure 3:**
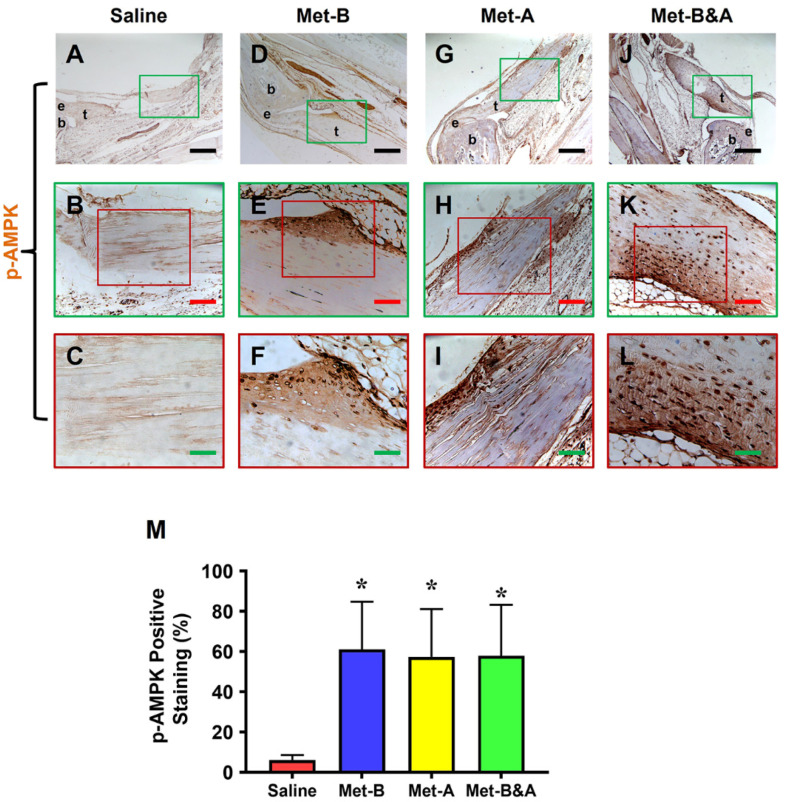
**Met injection activates AMPK.** IHC staining revealed elevated p-AMPK levels in the wound area of mice injected with Met (indicated by brown areas, (**D**–**L**)) in contrast to the minimal p-AMPK staining observed in the saline-injected group (**A**–**C**). Semi-quantification results indicated that over 57% of tendon cells in mice treated with Met injection exhibited positive p-AMPK staining. Conversely, less than 6.2% of tendon cells in mice receiving saline injection showed positive p-AMPK staining (**M**). * *p* < 0.01 compared to saline. Each of the small boxes, presented in green (**A**,**D**,**G**,**J**) is correspondingly enlarged in larger boxes (**B**,**E**,**H**,**K**). Each of the small boxes, presented in red (**B**,**E**,**H**,**K**) is correspondingly enlarged in larger boxes (**C**,**F**,**I**,**L**). Black bars: 500 µm; red bars: 200 µm; green bars: 50 µm. **Met-B:** Met-Before; **Met-A**: Met-After; **Met-B&A**: Met-Before-and-After; **t**: tendon; **b**: bone; **e**: enthesis.

Furthermore, our investigation demonstrated elevated levels of phosphorylated AMPK (p-AMPK) in the tendons of mice treated with IP Met injection (Figure 3D–L), while minimal p-AMPK staining was observed in saline-injected mouse tendons (Figure 3A–C). The semi-quantification indicated that more than 57% of tendon cells in the Met-injected mice were positively stained for p-AMPK, compared to only 6.1% for positively stained tendon cells in the saline-injected group (Figure 3M).

### 2.3. Met Injection Reduces TGF-β1 Levels

**Figure 4:**
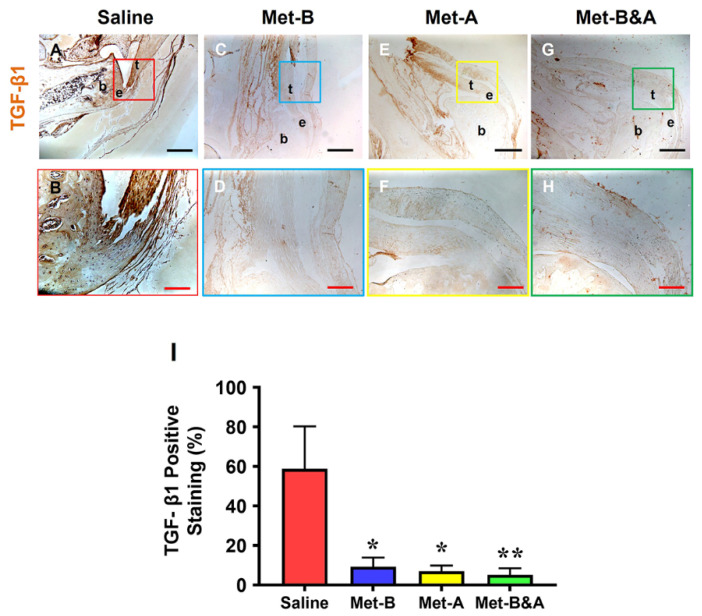
**Met injection decreases TGF-β1 levels.** The IHC results showed that TGF-β1 levels were increased in the wound area of saline-injected mice (**A**,**B**). However, the levels of TGF-β1 were decreased in the wounded tendons of mice with Met injection (**C**–**H**) compared to the saline-treated group. Semi-quantification results revealed that over 58% of tendon cells in the saline group displayed positive staining for TGF-β1. In contrast, approximately 9.2% of cells in the Met-B group, 7.0% in the Met-A group, and 5.2% in the **Met-B&A** group exhibited positive staining for TGF-β1 (**I**). * *p* < 0.05 compared to saline; ** *p* < 0.05 compared to saline and **Met-B**. Each of the small boxes, presented in four different colors (**A,C,E,G**) is correspondingly enlarged in larger boxes (**B,D,F,H**). Black bars: 500 µm; red bars: 200 µm. **Met-B**: Met-Before; **Met-A**: Met-After; **Met-B&A**: Met-Before-and-After; **t**: tendon; **b**: bone; **e**: enthesis.

High levels of TGF-β1 were detected in the tendon area of mice subjected to saline injection (Figure 4A,B). Conversely, the tendon tissues of mice in all three IP Met-injected groups exhibited significantly lower levels of positive staining for TGF-β1 (Figure 4C–H). The semi-quantitative analysis indicated that over 58% of tendon cells in the saline group were positively stained with TGF-β1. In contrast, approximately 9.2% of cells in the **Met-B** group, 7.0% in the Met-After group, and 5.2% in the Met-Before-and-After group displayed positive staining for TGF-β1 (Figure 4I).

### 2.4. Met Injection Inhibits α-SMA^+^ Cell Migration

**Figure 5:**
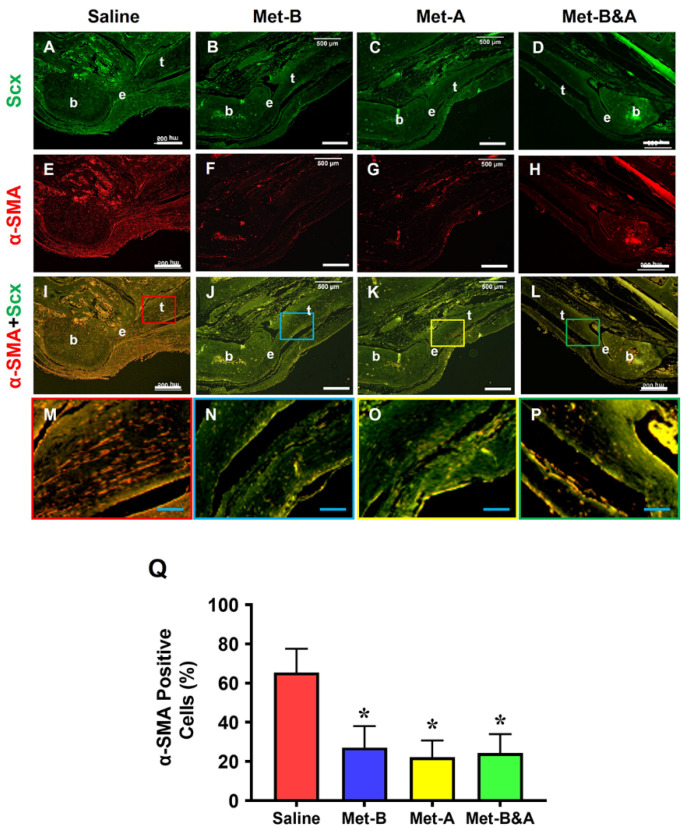
**Met injection inhibits α-SMA^+^ cell migration.** The histological analysis of mouse tendon tissues showed a lot of α-SMA^+^-stained cells (red) in the saline-treated group (**E**,**I**,**M**). However, a smaller number of α-SMA^+^-stained cells was found in the wounded tendons of mice with Met injections (**F**–**P**) compared to the saline-treated group. There was no significant difference in Scx^+^ cells (green fluorescent cells) in the wound areas of all four groups (**A**–**D**). Semi-quantification results indicated that over 65% of α-SMA^+^-stained cells were present in the tendon tissues in the saline group. However, 27.1% of the cells in the **Met-B** group, 22.4% of the cells in the **Met-A** group, and 24.26% of the cells in the **Met-B&A** group were positively stained for α-SMA (**Q**). * *p* < 0.01 compared to saline. Each of the small boxes, presented in four different colors (**I**–**L**) is correspondingly enlarged in the larger boxes (**M**–**P**). White bars: 500 µm; blue bars: 125 µm. **Met-B**: Met-Before; **Met-A**: Met-After; **Met-B&A**: Met-Before-and-After; **t**: tendon; **b**: bone; **e**: enthesis.

The tissue sections from the wounded Achilles tendons of the mice exhibited numerous α-SMA^+^ cells (red in Figure 5E,I,M). Met injection inhibited the migration of α-SMA^+^ cells (red fluorescent cells) in all three treated groups (Figure 5F–P). There was no significant difference in the number of Scx^+^ cells (green fluorescent cells) in the wound areas across all four groups (Figure 5A–D). The semi-quantitative analysis revealed that 65% of the red fluorescent cells in the tendons of the saline group were located within the tendon tissues. However, 27.1% of the cells in the tendons of the **Met-B** group, 22.4% in the **Met-A** group, and 24.3% in the **Met-B&A** group exhibited red fluorescence (Figure 5Q).

**Figure 6:**
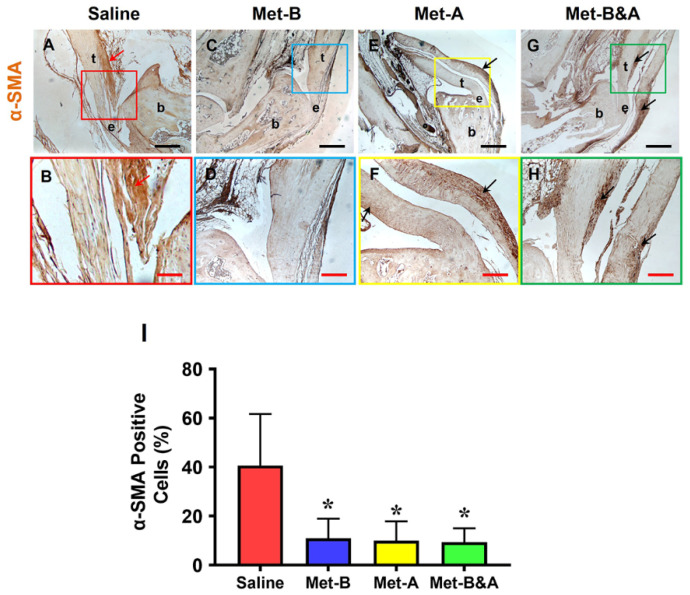
**Met injection inhibits α-SMA^+^ cell migration**. The IHC staining shows that α-SMA^+^ cells (positively stained with brown) were increased in the wound area of saline-injected mice (red arrow, (**A**,**B**)). There were few α-SMA^+^ cells in the **Met-B**-injected group (**C**,**D**), and only minimal α-SMA^+^ cells were stained in the paratenon of the tendons treated with Met in the other two groups (black arrows; (**E**–**H**)). Semi-quantification results indicated that 40.6% of the cells in the saline-treated mice were positively stained with α-SMA. However, 10.9% of the cells in the **Met-B** group, 10% of the cells in the **Met-A** group, and 9.4% of the cells in the **Met-B&A** group were positively stained with α-SMA (**I**). * *p* < 0.01 compared to saline. Each of the small boxes, presented in four different colors (**A,C,E,G**) is correspondingly enlarged in the larger boxes (**B,D,F,H**). Black bars: 500 µm; red bars: 200 µm. **Met-B**: Met-Before; **Met-A**: Met-After; **Met-B&A**: Met-Before-and-After; **t**: tendon; **b**: bone; **e**: enthesis.

Further analysis through immunostaining revealed that Met injection inhibited α-SMA^+^ cell migration. High concentrations of α-SMA^+^ cells were identified in the tendon tissues of mice treated with IP saline injection (red arrows indicate brown areas in Figure 6A,B). No α-SMA^+^ cells were detected in the tendon tissues of the **Met-B**-injected group (Figure 6C,D).

In the paratenon of the tendons, minimal α-SMA^+^ cell staining was observed in mice treated with IP injections of **Met-A** (black arrows in Figure 6E,F) and **Met-B&A** (black arrows in Figure 6G,H). The semi-quantification results indicated that 40.6% of the cells in the saline-treated mice were positively stained with α-SMA. In contrast, approximately 10.9% of cells in the **Met-B** group, 10% in the **Met-A** group, and 9.4% in the **Met-B&A** group exhibited positive staining for α-SMA (Figure 6I).

### 2.5. Met Injection Improves Healing Quality of Wounded Tendons

**Figure 7:**
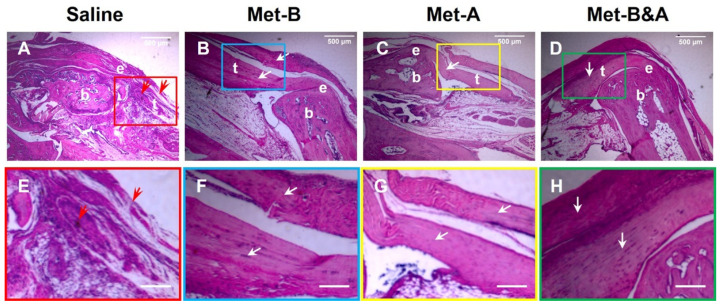
**Met injection inhibits scar tissue formation.** H&E staining of wounded tendon tissue sections at 4 weeks post-surgery showed scar tissue with high-density cells and poorly organized collagen fibers in the saline-treated mouse tendon (red arrows in (**A**,**E**)), while the tendon structure was well-organized in the Met injection groups (white arrows, (**B**–**D**,**F**–**H**)). Each of the small boxes, presented in four different colors (**A**–**D**) is correspondingly enlarged in the larger boxes (**E**–**H**). Bars: 500 µm. **Met-B**: Met-Before; **Met-A**: Met-After; **Met-B&A**: Met-Before-and-After; **t**: tendon; **b**: bone; **e**: enthesis.

The cell density in the wound area of the mice treated with IP saline injection (red arrows in Figure 7A,E) was greater than the three Met injection groups, which showed some presence of inflammatory cells (giant cells in Figure 7B–D). The tendon structure in the saline-treated mice was characterized by thin, loosely arranged collagen fibers (red box area in Figure 7A). In contrast, the cells in the tendons of the IP Met-injected mice exhibited a normal-looking elongated shape (white arrows in Figure 7B–D,F–H). The collagen fibers in these tendons were well-organized (boxes in Figure 7B–D).

### 2.6. Met Injection Inhibits Scar Formation by Decreasing Loose Collagen Fibers, Collagen Type III, and Increasing Collagen Type I

**Figure 8:**
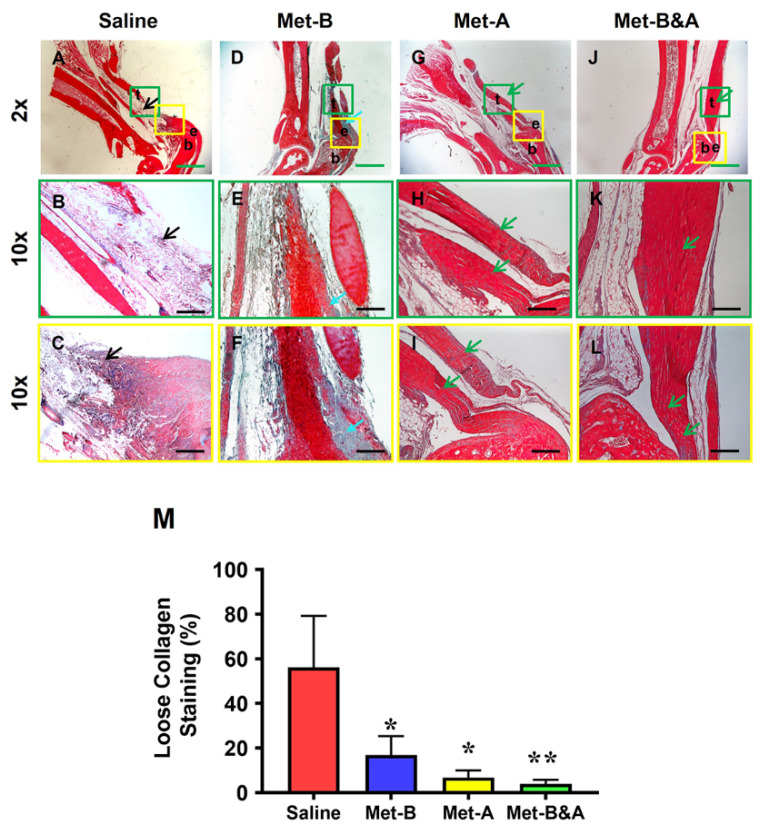
**Met inhibits scar tissue formation by decreasing loose collagen fibers.** Masson’s trichrome staining of tendon tissues showed loose collagen fibers stained with blue in the saline group (black arrow, (**A**–**C**)), while the Met-treated groups showed dense collagen fibers (**D**–**L**) with some loose collagen fibers in the Met-B group (blue arrow, (**D**–**F**)). Longer treatment durations of Met (**Met-A** and **Met-B&A**) showed improved wounded tendon healing as evidenced by more positive collagen I staining (green arrows; (**G**–**L**)). Semi-quantification results showed that 56% of the cells in saline-treated tendons were positively stained with loose collagen fibers. However, about 17% of the cells in **Met-B**, 6.8% of the cells in **Met-A**, and 4% of the cells in **Met-B&A** were positively stained with loose collagen (**M**). * *p* < 0.01 compared to saline; ** *p* < 0.05 compared to **Met-B** and **Met-A**. Each of the small boxes, presented in green (**A,D,G,J**) is correspondingly enlarged in the larger boxes (**B,E,H,K**). Each of the small boxes, presented in yellow (**A,D,G,J**) is correspondingly enlarged in the larger boxes (**C,F,I,L**). Green bars: 1 mm; black bars: 200 µm. **Met-B**: Met-Before; **Met-A**: Met-After; **Met-B&A**: Met-Before-and-After; **t**: tendon; **b**: bone; **e**: enthesis.

At 4 weeks post-surgery, the healed tendon tissues in the wound areas of the mice treated with saline injection were loose and stained blue (black arrows in Figure 8A–C). In contrast, dense collagen fibers were observed in the tendons of mice receiving Met injection (Figure 8D–L). Although some loose collagen fibers were present in the **Met-B**-injected tendons, the quantity of blue-stained collagen fibers in this group (blue arrows in Figure 8D–F) was significantly less than in the saline injection group. Met injection enhanced wounded tendon healing, as evidenced by an increase in positive collagen I staining (green arrows in Figure 8D–L). Semi-quantification showed that 56% of the cells in the saline-treated tendons were positively stained with loose collagen fibers (Figure 8M). However, 17% of the cells in the tendon tissues of the mice in **Met-B**, 6.8% of the cells in the tendons in **Met-A**, and 4% of the cells in **Met-B&A** were positively stained with loose collagen (Figure 8M).

**Figure 9:**
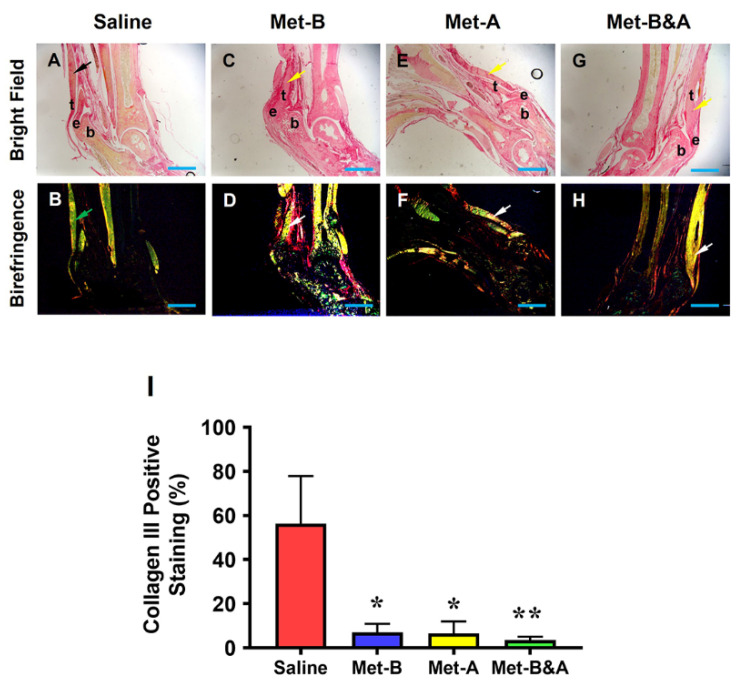
**Met inhibits scar tissue formation by decreasing collagen III levels and increasing collagen I levels.** Picro Sirius red staining of tendon tissues under light microscope and polarized light showed that healed tendon in the saline group was formed by collagen III (yellow in (**A**), black arrow; green in (**B**), green arrow, respectively). However, the healed tendon tissues in the Met injection groups were formed by collagen I (red in (**C**,**E**,**G**), yellow arrow; red and yellow in (**D**,**F**,**H**), white arrow). Semi-quantification results showed that more than 56% of the cells in the saline-treated tendons were positively stained with collagen III (**I**). However, about 7% of the cells in **Met-B**, about 6.56% of the cells in **Met-A**, and about 3.5% of the cells in **Met-B&A** were positively stained with collagen III (**I**). Bars: 1 mm. **Met-B**: Met-Before; **Met-A**: Met-After; **Met-B&A**: Met-Before-and-After; **t**: tendon; **b**: bone; **e**: enthesis. * *p* < 0.01 compared to saline; ** *p* < 0.05 compared to Met-A, and Met-B.

The results also revealed that the healed tendon in the saline group was predominantly formed by collagen III (arrow indicates yellow in Figure 9A; arrow indicates green in Figure 9B). In contrast, the healed tendon tissues in the Met injection groups were mainly composed of collagen I (arrows point to red in Figure 9C,E,G, and yellow in Figure 9D,F,H). Semi-quantification showed more than 56% of the cells in the saline-treated tendons were positively stained with collagen III (Figure 9I). However, about 7% of the cells in the tendon tissues of the mice in **Met-B** were positively stained with collagen III, about 6.6% of the cells in the tendons in **Met-A** were positively stained with collagen III, and about 3.5% of the cells in the tendons of the mice in **Met-B&A** were positively stained with collagen III (Figure 9I).

**Figure 10:**
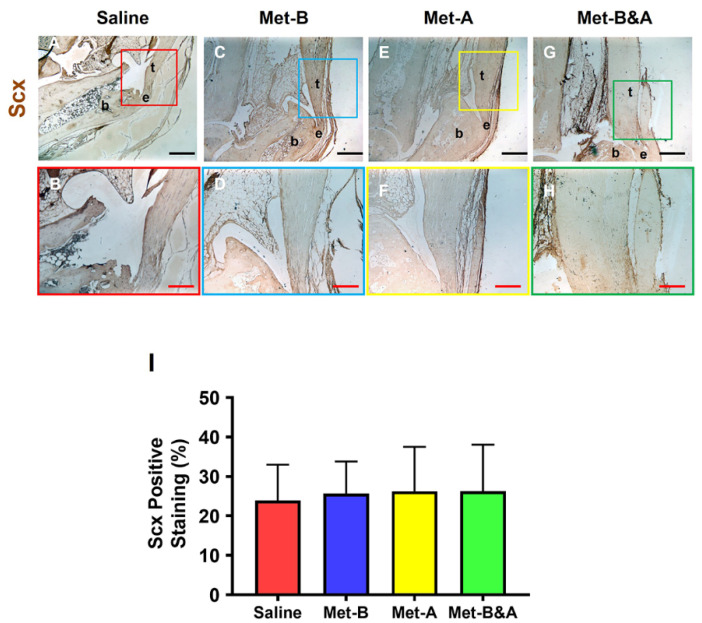
**Met does not change Scx^+^ cell numbers in mouse Achilles tendon tissues.** The Scx^+^ cell numbers did not show significant differences in the wound area between all groups treated either with saline injection (**A**,**B**) or with Met injection (**C**–**H**) by IHC staining. Semi-quantification results showed no significant differences in the Scx expression in all groups (**I**). Each of the small boxes, presented in four different colors (**A,C,E,G**) is correspondingly enlarged in the larger boxes (**B,D,F,H**). Black bars: 500 µm; red bars: 200 µm. **Met-B**: Met-Before; **Met-A**: Met-After; **Met-B&A**: Met-Before-and-After; **t**: tendon; **b**: bone; **e**: enthesis.

Moreover, there was no significant difference in Scx expression across all mouse tendons (Figure 10).

## 3. Discussion

This study showed that administering Met via IP injections before, after, or both before and after inducing Achilles tendon injuries effectively prevented the translocation of HMGB1 from nuclei to the extracellular matrix. Furthermore, Met administration was found to reduce serum HMGB1 levels, activate AMPK as evidenced by elevated p-AMPK levels, and decrease TGF-β1 expression. Additionally, Met inhibited the migration of α-SMA^+^ cells, diminished collagen type III accumulation, and augmented collagen type I production. These actions of Met collectively contributed to the attenuation of scar tissue formation in the injured tendons.

The results of this study suggest that Met possesses anti-inflammatory, pro-healing, and anti-fibrotic properties. This is the first piece of evidence to demonstrate the anti-fibrotic effects of Met on Achilles tendon injuries. Previous research indicated that Met prevents peritendinous fibrosis by inhibiting TGF-β1 signaling in a surgical rat model of flexor tendon peritendinous adhesion [37]. Our findings are consistent with the conclusions of that prior study.

This study demonstrated that Met suppressed the release of HMGB1 into the extracellular matrix of damaged tendons, thereby mitigating both systemic and localized inflammatory responses. It is well-established that HMGB1 is released from the nuclei of cells into the wound site upon tissue injury. The released HMGB1 then promotes the recruitment of inflammatory cells to the site of injury [38,39]. HMGB1’s role in the progression of chronic inflammatory diseases, including rheumatoid arthritis, cardiovascular disease, sepsis, and cancer, has been well-documented [40]. Our previous research has also indicated that in chronic Achilles tendon disorders, such as tendinopathies, HMGB1 release induced by stress or injury can initiate inflammatory pathways that lead to tissue degradation. Importantly, our studies have shown that targeting HMGB1 with Met can effectively mitigate these detrimental effects [17,18].

It is known that chronic inflammation can lead to fibrosis or scarring, characterized by the accumulation of excessive extracellular matrix components, including collagen. HMGB1, a potent mediator of inflammation, plays a significant role in various fibrotic diseases and thus represents a promising target for reducing inflammation and subsequent scarring. Its release following tissue damage has been implicated in the fibrotic processes observed in conditions such as systemic sclerosis [41], liver fibrosis [31], renal fibrosis [20], and pulmonary fibrosis [21]. In contrast to the scarless healing of fetal cutaneous wounds that occurs without inflammation, scar-forming fetal wounds exhibit increased HMGB1 levels, leading to a rise in fibroblasts, blood vessels, and macrophages, thus enhancing scar formation [22]. The pro-fibrotic effects of HMGB1 were also demonstrated in a rabbit model of hypertrophic scarring, where the external application of HMGB1 exacerbated scar formation. In contrast, its inhibition by Box A significantly reduced scarring [23]. Other studies reported that the suppression of HMGB1 using agents like glycyrrhizic acid and salvianolic acid B offered therapeutic benefits in the treatment of cardiac fibrosis and liver disease, respectively, in rat models [42,43]. Our study’s findings, which highlight the efficacy of using Met to inhibit HMGB1 and reduce tendon scarring, are in line with these earlier investigations.

Met, as an inhibitor of HMGB1, presents as a viable pharmaceutical candidate to reduce scarring and enhance tendon healing post-injury by attenuating inflammation. Our study has demonstrated that Met can inhibit HMGB1 release resulting from acute tendon injuries, which contributes to local and systemic inflammation—key factors in scar tissue development. Our previous work has also highlighted Met’s anti-inflammatory effects in an Achilles tendinopathy model characterized by HMGB1-related chronic inflammation. We showed that HMGB1 released into the tendon matrix triggers an inflammatory cascade, which was successfully inhibited by glycyrrhizic acid and Met, thereby reducing the associated inflammation and tissue degeneration [17,18]. The current study reinforces Met’s anti-inflammatory potential in the context of acute Achilles tendon injuries.

In addition to its anti-inflammatory benefits, Met also exhibits anti-fibrotic properties. Its effectiveness in mitigating fibrosis has been documented across various conditions, such as pulmonary [27,28], cardiac [29], joint capsular [30], and liver fibrosis [31], with evidence from both in vitro and in vivo studies. Met is known to dampen chronic inflammation and exerts direct anti-inflammatory effects via AMPK-dependent and independent pathways, consequently slowing down the fibrogenesis process [32,44].

The anti-fibrotic effect of Met is mediated via the activation of AMPK and interfering with the TGF-β1 signaling that mediates fibrogenic processes [45,46]. It was previously demonstrated that Met, by activating AMPK, provides protective effects against lung injury and impedes the progression of fibrosis in an animal model [47].

By activating AMPK, Met significantly counteracts fibrotic processes, primarily by interfering with TGF-β1 signaling, which is instrumental in the activation of fibroblasts to myofibroblasts—a critical step in fibrogenesis [27,46,47,48]. For example, in human idiopathic pulmonary fibrosis and a mouse model of bleomycin-induced lung fibrosis, AMPK activity was found to be diminished within fibrotic areas [27]. Remarkably, the pharmacological activation of AMPK by Met was shown to promote the resolution of fibrosis in an AMPK-dependent manner in the mouse model of lung fibrosis [27]. Consistent with these findings, our study corroborates that Met induces AMPK activation, as evidenced by elevated levels of phosphorylated AMPK, and reduces TGF-β1 concentrations, thereby inhibiting scar tissue formation in acutely injured tendons.

Our study has uncovered that administering Met to injured mouse tendons effectively hampers the recruitment of α-SMA^+^ myofibroblasts to the site of injury. Myofibroblasts, characterized by their prolific production of the extracellular matrix (ECM), are central to the pathology of fibrotic diseases due to their role in excessive ECM deposition [49]. These cells are identified as key producers of the potent fibrogenic cytokine TGF-β1 and are considered critical players in tissue fibrosis [50,51]. Concurrently, the activation of AMPK has been shown to suppress the differentiation of myofibroblasts induced by TGF-β1, suggesting a protective role for AMPK against the onset of fibrosis [52]. Met treatment, which stimulates AMPK signaling, thereby has the potential to attenuate myofibroblast differentiation via TGF-β1, reducing fibrotic lesion accumulation in conditions such as pulmonary fibrosis [53]. In the context of injury, TGF-β1 triggers tissue remodeling and fibrotic scar formation by promoting anabolic metabolism in activated myofibroblasts. Accordingly, our findings indicate that Met injections inhibit the formation of scar tissue in injured tendons by blocking the migration of α-SMA-expressing myofibroblasts.

Currently there are no effective approaches to promote scarless tendon healing. Physical therapy and surgery are the two primary approaches in the management of tendon fibrosis; however, they are ineffective in restoring scarless tendon healing as they fail to directly address the underlying mechanism of fibrosis. Many pharmacological agents including over-the-counter supplements (e.g., quercetin, forskolin), prescription medications (e.g., losartan, atorvastatin), antibodies and small molecules (e.g., TGF-β antibodies, IL-1 inhibitor), and the local injection corticosteroids have been used to target fibrosis [54]. However, the safety profile and efficacy of all these agents remain to be demonstrated in tendons in more rigorous preclinical and clinical studies.

Met is already an FDA-approved drug for oral use, and its safety profile is well-established. Moreover, its efficacy in treating fibrosis has been demonstrated in numerous animal studies as described above. A nationwide cohort study reported improved clinical outcomes in terms of overall mortality and hospitalization in pulmonary fibrosis patients treated with Met [55], and the activation of AMPK with Met in the lung tissues of patients with pulmonary fibrosis displayed lower fibrotic activity [27]. Therefore, Met should be effective in managing tendon fibrosis in clinical settings, albeit with minimal side effects or complications, as compared to a surgical approach.

This study has several limitations. Firstly, it exclusively utilized female mice, leaving the impact of hormonal fluctuations on tendon healing with Met treatment unexplored. Future research will delve into this aspect. Secondly, this study did not evaluate the mechanical properties of tendons healed after Met treatment. Consequently, the extent of functional improvement in the tendons remains undetermined. However, given the significant enhancement in the tendon’s organization and structure, as illustrated in Figure 7, it is reasonable to infer that tendon function post-Met treatment would be substantially improved. Finally, the approach of using systemic Met injections in this study may cause side effects in human patients and is also less clinically feasible. Future studies will investigate alternative Met administration methods, such as localized injections, to mitigate these concerns.

## 4. Materials and Methods

### 4.1. Animals

The protocol for animal use was approved by the Institutional Animal Care and Use Committee (IACUC) of the University of Pittsburgh (protocol# 18083391). All animal experiments were performed according to the relevant guidelines and regulations.

The animals involved in this study were between 8–10 weeks old and weighed about 20 ± 2 g. They were housed at the Division of Laboratory Animal Resources (DLAR) at the University of Pittsburgh, where they were maintained under standard conditions, including a 12 h light/dark cycle. At the conclusion of the experiments, all animals were euthanized using carbon dioxide, in accordance with established euthanasia procedures.

### 4.2. Tendon Wound Healing Model

**Figure 11:**
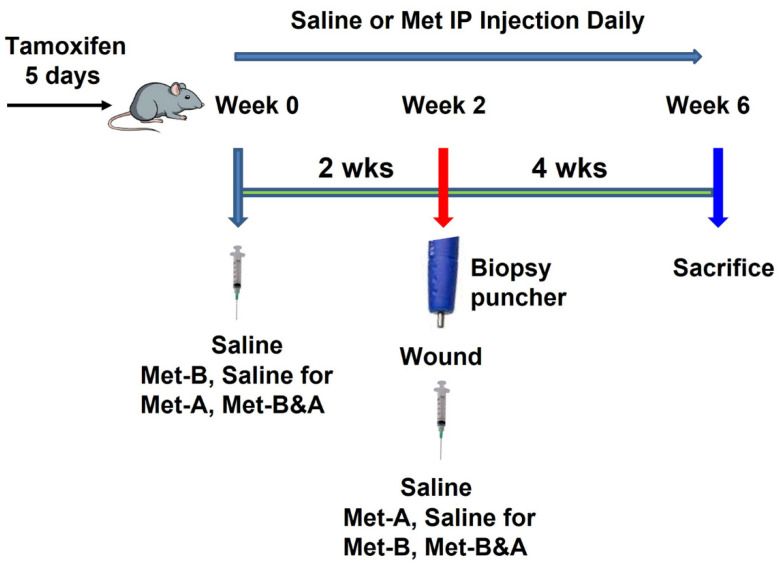
**Study Design Schematic**. **Saline** indicates IP injections of saline administered over a 6-week period. **Met-B** (Met-Before) denotes daily IP injections of Met for 2 weeks prior to tendon wounding. **Met-A** (Met-After) signifies daily IP injections of Met initiated post-tendon wounding and continued for 4 weeks. **Met-B&A** (Met-Before-and-After) describes the regimen of daily IP injections of Met starting 2 weeks before tendon wounding and extending for 4 weeks post-wounding. Met dosage: 160 mg/kg/day.

The tamoxifen-inducible α-SMA-CreERT2 mice were crossed with Scx-GFP mice, and then crossed with Ai9 Cre reporter mice to generate triple transgenic α-SMA-Ai9-Scx-GFP mice according to the published protocol, with some modifications [56]. Five consecutive daily IP injections of tamoxifen (100 mg/kg) were administered to the α-SMA-Ai9-Scx-GFP female mice, aged 10 weeks, prior to any intervention. Animals were divided into 4 groups with 6 mice in each group and administered with saline or Met (160 mg/kg) via IP injection daily for 2 weeks. Following this, a window defect was created in the Achilles tendon of all animals using a biopsy punch with a diameter of 0.5 mm. All animals were further injected with saline or Met for the next 4 weeks (Figure 11).

Mice in group 1 received an IP injection of saline daily for 6 weeks (**Saline**); those in group 2 were given an IP injection of Met (160 mg/kg/day) daily for 2 weeks before wounding, followed by IP injection of saline for 4 weeks after wounding (**Met-B**); the mice in group 3 received IP injections of saline for 2 weeks before wounding and were then administrated Met (160 mg/kg/day) via IP injection for 4 weeks after wounding (**Met-A**); the mice in group 4 received IP injections of Met (160 mg/kg/day) for 6 weeks (2 weeks before wounding and 4 weeks after wounding; **Met-B&A**). All mice were sacrificed 30 days post-wounding. Blood was collected from the left ventricle of the mouse using a 26-gauge needle immediately after euthanasia. In addition, the hind legs including the Achilles tendon and heel were harvested from all animals for further analysis.

### 4.3. Measurement of HMGB1 in Mouse Serum by ELISA

The HMGB1 concentrations in mouse serum samples were assessed using the ELISA kit, following the manufacturer’s instructions (Cat. #NBP2-62767; Novus Biologicals; Centennial, CO, USA). Blood samples were collected from the hearts of mice and allowed to sit at room temperature for 1 h prior to centrifugation at 1000× *g* for 15 min. The supernatant was then separated from the red blood cell pellets and stored at 2–8 °C for 7 days, at −20 °C for 30 days, or at −80 °C for 90 days if the HMGB1 levels were not immediately determined.

### 4.4. Histochemical Staining for the Structural Analysis of Mouse Tendon Tissues

The hind legs, including the Achilles tendon and heel, were dissected from the mice and fixed with 4% paraformaldehyde overnight at 4 °C. These fixed mouse legs were then decalcified using Formical-4 decalcifier (SKU # 1214-1 GAL; StatLab, McKinney, TX, USA), with the decalcifying solution changed every three days until the decalcification was completed. Following this, the decalcified mouse leg tissue was embedded in paraffin and sectioned into 5 μm-thick slices. These tissue sections were subjected to structural analysis with hematoxylin and eosin (H&E), and Masson’s trichrome staining, according to the standard protocols. The stained tissue sections were subsequently examined under a light microscope (Nikon eclipse, TE2000-U).

### 4.5. Picro Sirius Red Staining and Polarized Light Microscopy of Mouse Tendon

The paraffin-embedded mouse leg tissue block was cut into 5 μm-thick slices and stained with a Picro Sirius red kit (Cat. #ab150681, Abcam, Waltham, MA, USA) following the manufacturer’s protocol. These stained tendon tissue sections were then examined under a polarized light microscope (Nikon).

### 4.6. Analysis of Cell Distribution on Mouse Tissue Sections Using Fluorescent Microscopy

For the analysis of cell distribution, the decalcified mouse leg tissue samples were promptly immersed in O.C.T compound (Sakura Finetek USA Inc., Torrance, CA, USA) within disposable molds and frozen at −80 °C. Subsequently, cryostat sectioning was performed at −25 °C to yield approximately 5 µm-thick tissue sections, which were then left at room temperature overnight. The α-SMA^+^ cells and Scx^+^ cells in the tendon tissue sections were identified by red and green fluorescence, respectively, under a fluorescent microscope.

### 4.7. Immunohistochemistry (IHC) Staining on Mouse Tissue Sections

For IHC staining, the decalcified mouse leg tissue samples were prepared as above. Cryostat sectioning was performed on the frozen tissue samples to obtain about 5 µm-thick tissue sections, which were left at room temperature overnight. The tissue sections were fixed in 4% paraformaldehyde for 15 min and then the sections were incubated overnight at 4 °C with rabbit anti-α-SMA antibody (1:500; Cat. #ab124964, Abcam; Waltham, MA, USA), rabbit anti-TGF-β1 antibody (1:500; Cat. #ab215715, Abcam; Waltham, MA, USA), rabbit anti-Scx antibody (1:500; Cat. #ab58655, Abcam, Waltham, MA, USA), or rabbit anti-HMGB1 antibody (1:330; Cat. #ab18256, Abcam; Waltham, MA, USA). The positively stained results were tested using a rabbit-specific HRP/DAB IHC detection kit (Cat. #ab236466; Abcam, Waltham, MA, USA).

For AMPK activation testing, the fixed tissue sections were treated with 0.1% Triton X-100 at 37 °C for 30 min, then washed with PBS 3 times. The treated tissue sections were incubated with rabbit anti-AMPK antibody (1:500, Cat. #MA5-15815, ThermoFisher Scientific; Waltham, MA, USA) or with rabbit anti-phospho-AMPK antibody (1:500, Cat. #ab133448, Abcam; Waltham, MA, USA) at 4 °C overnight. The staining results were further tested using a rabbit-specific HRP/DAB IHC detection kit (Cat. #ab236466; Abcam, Waltham, MA, USA).

### 4.8. Semi-Quantitative Assessment of Stained Tendon Tissue and Cells

We followed a published protocol for performing semi-quantification of the staining results in tissues and cells [57]. Briefly, three random images were captured from each tendon tissue section using a Nikon Eclipse TE2000-U fluorescent microscope to obtain semi-quantitative staining results. In total, nine images were analyzed for each group, representing three sections from three mice. The areas with positive staining in the tissue sections were identified manually by inspecting the captured images and were subsequently processed using SPOT imaging software v5.3 (Diagnostic Instruments). The percentage of positive staining was calculated as follows: percentage of positive staining = (positively stained area/total area observed) × 100. The percentage of positive staining for each group was determined by averaging the resulting values. Note that the images taken at 10× magnification were utilized to analyze staining outcomes at the wound site, covering an area of roughly 0.25 mm^2^.

### 4.9. Statistical Analysis

All statistical analyses were conducted using GraphPad Prism (v7.03). A one-way ANOVA, followed by Fisher’s LSD test, was utilized for statistical comparison. A *p*-value < 0.05 between two groups was deemed to indicate a significant difference.

## 5. Conclusions

Our study reveals that Met has anti-fibrotic effects and can reduce the formation of scar tissue in injured tendons through IP injections. Thus, we suggest that Met could be employed as a singular therapeutic intervention for reducing scar tissue in tendon injuries. It holds the potential for direct application in clinical settings to improve the healing outcomes of tendon injuries and possibly other musculoskeletal tissue injuries.

## Data Availability

Data are contained within the article.
